# Factors associated with COVID-19 vaccine confidence among primary care providers in Kazakhstan, March–April 2021

**DOI:** 10.3389/fpubh.2023.1245750

**Published:** 2023-09-07

**Authors:** Dilyara Nabirova, Roberta Horth, Lena Kassabekova, Alden Henderson, Aizhan Yesmagambetova, Sevak Alaverdyan, J. Pekka Nuorti, Manar Smagul

**Affiliations:** ^1^Division of Global Health Protection in Central Asia, United States Centers for Disease Control and Prevention, Almaty, Kazakhstan; ^2^Central Asia Field Epidemiology Training Program, Asfendiyarov Kazakh National Medical University, Almaty, Kazakhstan; ^3^Health Sciences Unit, Faculty of Social Sciences, Tampere University, Tampere, Finland; ^4^Scientific and Practical Center of Sanitary-Epidemiological Examination and Monitoring, Branch of the National Center for Public Health, Almaty, Kazakhstan; ^5^Division of Global Health Protection, United States Centers for Disease Control and Prevention, Atlanta, GA, United States; ^6^Ministry of Healthcare, Astana, Kazakhstan; ^7^Manoogian Simone College of Business and Economics, American University of Armenia, Yerevan, Armenia; ^8^Infectious Diseases and Vaccinations Unit, Department of Health Security, Finnish Institute for Health and Welfare (THL), Helsinki, Finland

**Keywords:** COVID-19, vaccine confidence, primary care providers, attitude toward vaccination, childhood vaccines, Kazakhstan

## Abstract

**Introduction:**

Vaccination is a critical public health intervention, and vaccine hesitancy is a major threat. Globally, confidence in COVID-19 vaccines has been low, and rates of routine immunizations decreased during the COVID-19 pandemic. Because healthcare providers are a trusted source of information on vaccination in Kazakhstan, it was vital to understand their knowledge, attitudes and practices (KAP) related to both routine and COVID-19 vaccines.

**Methods:**

From March to April 2021, we conducted a cross-sectional study among the healthcare providers responsible for vaccination in 54 primary care facilities in three cities in Kazakhstan. All consenting providers anonymously completed structured online questionnaires at their place of work. A provider was classified as having COVID-19 vaccine confidence if they planned to get a COVID-19 vaccine, believed that COVID-19 vaccines are important to protect their community and either believed the vaccine was important to protect themselves or believed that getting a vaccine was safer than getting COVID-19. Statistical analysis included chi-square, Spearman’s rank correlation coefficient, and Poisson regression.

**Results:**

Of 1,461 providers, 30% had COVID-19 vaccine confidence, 40% did not, and 30% would refuse vaccination. Participants were mostly female (92%) and ≤ 35 years old (57%). Additionally, 65% were nurses, 25% were family physicians, and 10% were pediatricians. Adequate KAP for routine vaccines was low (22, 17, and 32%, respectively). Adequate knowledge was highest among pediatricians (42%) and family physicians (28%) and lowest among nurses (17%). Misconceptions about vaccines were high; 54% believed that influenza vaccines cause flu, and 57% believed that there is a scientifically proven association between vaccination and autism and multiple sclerosis. About half (45%) of the practitioners felt confident answering patient vaccine-related concerns. In adjusted models, COVID-19 vaccine confidence was positively associated with adequate knowledge of vaccines (prevalence ratio: 1.2, 95% confidence interval: 1.0–1.4) and adequate attitudes related to routine vaccines (3.1, 2.7–3.6).

**Conclusion:**

Our study uncovers critical areas for interventions to improve KAP related to routine immunizations and COVID-19 vaccine confidence among providers in Kazakhstan. The complex relationship between KAP of routine vaccines and COVID-19 vaccine confidence underscores the importance of addressing vaccine hesitancy more broadly and not focusing solely on COVID-19.

## Introduction

Vaccines are one of the most successful public health interventions. They contributed to eradication of smallpox, near-elimination of poliomyelitis, reduction of incidence of several vaccine-preventable diseases, increased population lifespan, and saved millions of lives annually (1–4). Vaccines are most effective when global coverage is high. Recent drops in global immunization rates threaten to reverse progress.

The World Health Organization (WHO) declared vaccine hesitancy one of the top 10 global health threats in 2019, before the COVID-19 pandemic began. Factors influencing vaccine hesitancy are complex, but the mass quantities of false and misleading information play an important role. Misinformation amplifies vaccine hesitancy and contributes to the loss of life and reduced quality of life for millions of people, both vaccinated and unvaccinated (5–7).

Primary healthcare providers play a key role in informing people about vaccines, encouraging them to vaccinate, and keeping vaccine coverage high in their populations (8–11). Primary healthcare providers’ knowledge, attitude, and beliefs toward vaccination influence their own immunization and can impact patients’ vaccine acceptance and increase vaccine uptake. Studies show wide variation in vaccine confidence among providers. Many providers had insufficient knowledge about the safety and effectiveness of vaccines or lacked time and interpersonal skills to persuade patients to vaccinate (12–14).

In Kazakhstan, coverage for childhood immunizations has decreased in recent years, and annually, approximately 5,000 of 360,000 infants are not vaccinated because of parental/caregiver refusal (15). As in many other countries, primary healthcare providers in Kazakhstan are a trusted source of vaccine information for the population (11). However, little is known about primary care providers’ views toward vaccines, including their knowledge, attitude, beliefs, and practices, and their ability to advocate for childhood vaccination.

Primary care providers in Kazakhstan have a large patient load. Each physician has 1,500–5,000 patients in their catchment area. Physicians are responsible for disease management programs, including tuberculosis diagnosis and management and administration of vaccination, patient education and other tasks (16). They play a critical role in promoting vaccination by providing accurate information about vaccines, addressing concerns and misconceptions, and administering vaccines to patients.

Over the last decade, national guidelines for routine immunization have been updated four times due to the introduction of new vaccines and changes in the immunization schedule (17–20). Nine childhood and eight adult vaccines against 20 infectious diseases are included in the national vaccine schedule. These vaccines are administered free of charge to patients at public and private primary care facilities. Flu vaccines are also free of charge to high-risk populations, including healthcare providers and children.

COVID-19 vaccines first became available in Kazakhstan in February 2021. They are free of charge. These vaccines had been developed in Kazakhstan and Russia, and there were no publicly available data from clinical trials on the efficacy of these vaccines at the time. Misinformation quickly ensued. Healthcare providers need to be well informed about these vaccines to counter increasing vaccine refusals. Understanding primary healthcare provider confidence in and knowledge about COVID-19 vaccines is crucial.

Therefore, we aimed to (1) determine levels of vaccine confidence among providers toward COVID-19 vaccination; (2) describe knowledge, attitude, and practice toward vaccination, including routine childhood, influenza, and COVID-19 vaccines; (3) assess factors associated with COVID-19 vaccine confidence; and (4) determine the correlation between COVID-19 vaccine confidence and knowledge, attitude, and practice toward childhood and influenza vaccination.

## Materials and methods

### Study design

We conducted a cross-sectional study among primary healthcare providers in 23 public and 31 private primary care facilities in three geographically dispersed cities: Shymkent (population: 1.1 M), Turkestan (population: 186 K), and Aktobe (population 518 K). These cities were selected because they were considered cities at greatest risk of vaccine preventable disease outbreak and had the highest reported incidence of measles in the country in 2019–2020 despite measles vaccination coverage >95% (15).

### Study participants

Participants included all family physicians, pediatricians, and nurses responsible for administrating vaccines who were at their workplace during the study. In each primary care facility, a physician usually works with at least two nurses. Only providers who provided written informed consent were interviewed in three cities between March and April 2021. No personally identifiable information on providers or the title of the primary care facility was collected during the interviews.

### Survey tool

We used an anonymous self-administered structured questionnaire in Kazakh and Russian languages. Seven residents of the Central Asia Frontline and Advanced Field Epidemiology Training Program (FETP) piloted the questionnaire on a small group of providers, revised the questionnaire based on observations and comments of the interviewed piloted group, and then interviewed study participants. Data from the pilot were not included in the final dataset.

We estimated the internal consistency of each question of the questionnaire using Cronbach’s alpha coefficient (21). The internal consistency for the 107 questions was 0.899. The average survey length was 20 min.

The survey tool included sociodemographic questions and questions related to vaccine knowledge, attitudes, and practices (Supplementary Tables 1A–D):

- Knowledge (K) was accessed using 26 questions. Each knowledge question was scored as “1-correct,” “0-incorrect,” or “0-difficult to answer.”
- Attitudes (A) were assessed using 55 questions on a five-point Likert scale, where “absolutely disagree,” “disagree,” “indifferent,” “agree,” and “absolutely agree,” were scored as 1, 2, 3, 4, and 5, respectively.
- Practices (P) were assessed using 14 questions on a three-point Likert scale, where “2 = always,” “1 = sometimes,” and “0 = never” were rated as 2, 1, and 0, respectively.

### Outcomes of interest

The survey tool includes four questions to assess confidence in COVID-19 (22, 23):

1. Do you plan to get the COVID-19 vaccine?
2. Do you believe that the COVID-19 vaccine is important to protect your community?
3. Do you believe that the COVID-19 vaccine is important to protect yourself?
4. Do you believe that the COVID-19 vaccine is safer than getting COVID-19?

Answers to these questions were categorized as follows:

- Vaccine refusal: no to all questions were classified as having vaccine refusal (24).
- Vaccine confidence: affirmative responses to questions 1 and 2 and at least 3 or 4 (24–26).
- Vaccine hesitant: not being in any of the two above categories.

Individual respondents’ correct scores for knowledge, attitudes, or practices were summed in each domain. Individual KAP scores for each respondent were then dichotomized as adequate and not adequate KAP for each domain with scores 70% or above as the cut-off (27, 28).

### Ethical information

The study was approved by the Ministry of Healthcare of Kazakhstan and the institutional review boards of the Kazakhstan Graduate School of Public Health. This activity was reviewed by the CDC and was conducted consistently with applicable U.S. federal law and CDC policy.^1^

### Statistical analysis

Data cleaning and analysis were conducted in R version 4.2.1 (R Foundation for Statistical Computing, Vienna, Austria). We accessed the power of our study to detect statistically significant differences (29–31) (Supplementary Figure 1). To assess associations with adequate KAP and vaccine confidence, we used the chi-square test for categorical variables and the Mann–Whitney U test for continuous variables. We used Poisson regression to calculate prevalence ratios (PRs) and 95% confidence intervals (CIs) for variables independently associated with COVID-19 vaccine confidence and adequate KAP.

We used Spearman’s rank correlation coefficient to measure the correlation between KAP variables and COVID-19 vaccine confidence (32). We also assessed the adjusted relationship between these variables using multivariable Poisson models and present adjusted prevalence ratios (aPR). Variables with a value of *p* < 0.05 in the bivariable analysis and possible confounders were selected for inclusion in multivariable Poisson regression models (33, 34) (Supplementary Table 2). We checked for multicollinearity using the generalized variance inflation factor (35, 36).

## Results

### Sociodemographic data and vaccine confidence

Of the 3,500 providers employed at the studied primary care facilities, 42% were on-site and available at the time of the study and responded to the survey (69% in Aktobe, 33% in Shymkent, and 28% in Turkestan). Of the 1,461 participants, 951 were nurses (65%), 360 (25%) were family physicians, and 150 (10%) were pediatricians. Among the participants, 1,351 (92%) were female, 832 (57%) were 35 years old or younger, 692 (47%) were from Aktobe, 489 (34%) were from Shymkent, and 280 (19%) were from Turkistan (Table 1).

**Table 1:** Perceptions toward COVID-19 vaccination among primary care providers, Aktobe, Shymkent, and Turkestan cities, Kazakhstan, 2021 (*N* = 1,461).

Characteristics	Vaccine confidence *n* = 435 (30%^a^)	Vaccine hesitancy *n* = 587 (40%^a^)	Vaccine refusal *n* = 439 (30%^a^)	Total *N* = 1,461 (100%^b^)	*p* value
Occupation					
Nurse	276 (29)	382 (40)	293 (31)	951 (65)	0.202
Family physician	115 (32)	134 (37)	111 (31)	360 (25)	
Pediatrician	44 (29)	71 (47)	35 (23)	150 (10)	
Gender					
Male	24 (22)	44 (40)	42 (38)	110 (8)	0.076
Female	411 (30)	543 (40)	397 (29)	1,351 (92)	
Age, years					
18–26	84 (25)	150 (44)	108 (32)	342 (23)	0.005
27–35	140 (29)	186 (38)	164 (33)	490 (34)	
36–66	204 (35)	231 (39)	154 (26)	589 (40)	
Missing	7 (18)	20 (50)	13 (33)	40 (3)	
City of residence					
Shymkent	151 (31)	190 (39)	148 (30)	489 (34)	<0.001
Aktobe	232 (34)	266 (38)	194 (28)	692 (47)	
Turkestan	52 (19)	131 (47)	97 (35)	280 (19)	
Professional experience, years					
Median [Min, Max]	10 [1, 44]	7 [1, 46]	6 [1, 41]	8 [1, 46]	<0.001
0–10	236 (26)	376 (41)	295 (33)	907 (62)	
11–20	97 (36)	88 (33)	82 (31)	267 (18)	
21 or more	102 (36)	123 (43)	62 (22)	287 (20)	
Child in the family					
Yes	324 (32)	397 (39)	300 (29)	1,021 (70)	0.043
No	111 (25)	190 (43)	139 (32)	440 (30)	
Number of children in the family					
Median [Min, Max]	2 [1, 6]	2 [1, 7]	2 [1, 6]	2 [1, 7]	0.938
1–2	168 (31)	219 (40)	157 (29)	544 (37)	
3 or more	156 (33)	178 (37)	143 (30)	477 (33)	
Missing	111 (25)	190 (43)	139 (32)	440 (30)	
Older adult in the household					
Yes	104 (32)	122 (38)	99 (30)	325 (22)	0.486
No	331 (29)	465 (41)	340 (30)	1,136 (78)	

^a^Row percent; ^b^Column percent.

We found that 435 (30%) had COVID-19 vaccine confidence, and 439 (30%) providers would refuse to receive the vaccine. The proportion with vaccine confidence differed significantly by age, city, work experience, and having children in the family. Confidence was 35% among providers 36 years old and above and 25% among those 18–26 years old.

### Knowledge about vaccination

Adequate knowledge about vaccination was 22%. It was higher among family physicians (28%) and pediatricians (42%) than among nurses (17%; *p* < 0.001; Table 2). Knowledge related specifically to vaccine contraindications was lowest among nurses, less than half could correctly identify vaccine contraindications, and highest among pediatricians. However, 44% of pediatricians, 54% of family physicians, and 55% of nurses incorrectly believed that influenza vaccine causes flu. Additionally, 50% of providers incorrectly believed that the pertussis vaccine causes sudden infant death syndrome. Just over half (57%) of providers incorrectly believed that there is a scientifically proven association between vaccination and autism and multiple sclerosis. A lower proportion of nurses (44%) than family physicians (56%) believed that COVID-19 vaccination was a justified prevention measure (*p* < 0.001).

**Table 2:** Correct answers to the question on knowledge toward vaccination among primary care providers, Aktobe, Shymkent, and Turkestan cities of Kazakhstan, 2021 (*N* = 1,461).

Characteristics	Total (*N* = 1,461)	Nurse (*n* = 951)	Family physician (*n* = 360)	Pediatrician (*n* = 150)	*p* value^a^
Had adequate vaccine knowledge (scored ≥ 70%)	327 (22)	163 (17)	101 (28)	63 (42)	<0.001
Temporary contraindications to immunization for all vaccines					
Fever, Yes (T)^b^	864 (59)	481 (51)	265 (74)	118 (79)	<0.001
Uncontrolled seizures or progressive encephalopathy, Yes (T)	847 (58)	475 (50)	253 (70)	119 (79)	<0.001
Acute diseases, regardless of temperature, Yes (T)	823 (56)	460 (48)	253 (70)	110 (73)	<0.001
Prematurity, Yes (T)	766 (52)	438 (46)	224 (62)	104 (69)	<0.001
Birth weight < 2,500 g, No (T)	717 (49)	451 (47)	171 (48)	95 (63)	0.001
Steroid use, Yes (T)	659 (45)	371 (39)	195 (54)	93 (62)	<0.001
Antibiotics use, No (T)	452 (31)	319 (34)	99 (28)	34 (23)	0.007
Permanent contraindications to immunization for all vaccines					
Severe allergic reaction to the previous dose, Yes (T)	866 (59)	496 (52)	256 (71)	114 (76)	<0.001
Malignant neoplasms with active chemotherapy, Yes (T)	817 (56)	458 (48)	240 (67)	119 (79)	<0.001
Immunodeficiency diseases, Yes (T)	808 (55)	453 (48)	241 (67)	114 (76)	<0.001
General knowledge about vaccination					
Pertussis vaccine causes sudden infant death syndrome, No (T)	726 (50)	474 (49)	174 (48)	78 (52)	0.743
The flu vaccine causes the flu, No (T)	672 (46)	423 (45)	165 (46)	84 (56)	0.031
It is necessary to restart the Hepatitis B vaccine series if a dose was missed or delayed, No (T)	665 (46)	445 (47)	154 (43)	66 (44)	0.396
Simultaneous administration of multiple vaccines causes chronic health problems (overload the immune system), No (T)	655 (45)	424 (45)	141 (39)	90 (60)	<0.001
There is a scientifically proven association between vaccination and autism, multiple sclerosis, No (T)	630 (43)	411 (43)	151 (42)	68 (45)	0.776
Children who had pertussis may be vaccinated later with a vaccine containing the pertussis component, Yes (T)	429 (29)	261 (27)	110 (31)	58 (39)	0.017
COVID-19 vaccination is an effective preventive measure, Yes (T)	806 (55)	490 (52)	221 (61)	95 (63)	<0.001
COVID-19 vaccination is a justified prevention measure, Yes (T)	699 (48)	417 (44)	201 (56)	81 (54)	<0.001
Efficacy of Sputnik V is >90%, Yes (T)	660 (45)	402 (42)	177 (49)	81 (54)	0.006
Sputnik V is a vector vaccine for COVID-19, Yes (T)	599 (41)	362 (38)	170 (47)	67 (45)	0.007

^a^Chi-square *p* value; ^b^T, true answer.

### Practices related to vaccination

The proportion with adequate practices related to vaccination differed by profession and was 29% among nurses, 33% among family physicians, and 42% among pediatricians (*p* = 0.010; Table 3). Additionally, 43% of nurses, 44% of family physicians, and 55% of pediatricians felt confident about answering their patients’ vaccine-related questions.

**Table 3:** Practices toward vaccination among primary care providers, Aktobe, Shymkent, and Turkestan cities of Kazakhstan, 2021 (*N* = 1,461).

Characteristics	Total (*N* = 1,461)	Nurse (*n* = 951)	Family physician (*n* = 360)	Pediatrician (*n* = 150)	*p* value^a^
Had adequate vaccine practice (scored ≥ 70%)	465 (32)	283 (29)	119 (33)	63 (42)	0.010
Relies on own judgment, not manufacturer’s recommendations when administering vaccines, Always^b^	514 (35)	338 (36)	131 (36)	45 (30)	0.173
Relies on colleagues’ opinions when administering vaccinations and working with those who refuse, Always	350 (24)	234 (25)	87 (24)	29 (19)	0.725
Recommends immunization according to friends and family members, Always	742 (51)	462 (49)	179 (50)	101 (67)	<0.001
Feels it is difficult to talk with parent/individuals about vaccinations, Never	531 (36)	336 (35)	134 (37)	61 (41)	0.344
Feels confident when answering patient questions about vaccines, Always	653 (45)	413 (43)	158 (44)	82 (55)	0.019
Feels comfortable addressing patient’s vaccine side effects concerns, Always	634 (43)	403 (42)	159 (44)	72 (48)	0.599
Always receives continuing education in the field of vaccination:	659 (45)	424 (45)	153 (43)	82 (55)	0.031
Independently studying scientific literature on vaccination	492 (34)	287 (30)	154 (43)	51 (34)	<0.001
Attends seminars and trainings held in medical organizations	652 (45)	426 (45)	147 (41)	79 (53)	0.047
Taking online trainings	526 (36)	335 (35)	129 (36)	62 (41)	0.143

^a^Chi-square *p* value; ^b^Always is one of the comparison categories of practice variables.

### Attitudes toward vaccination

**Figure 1:**
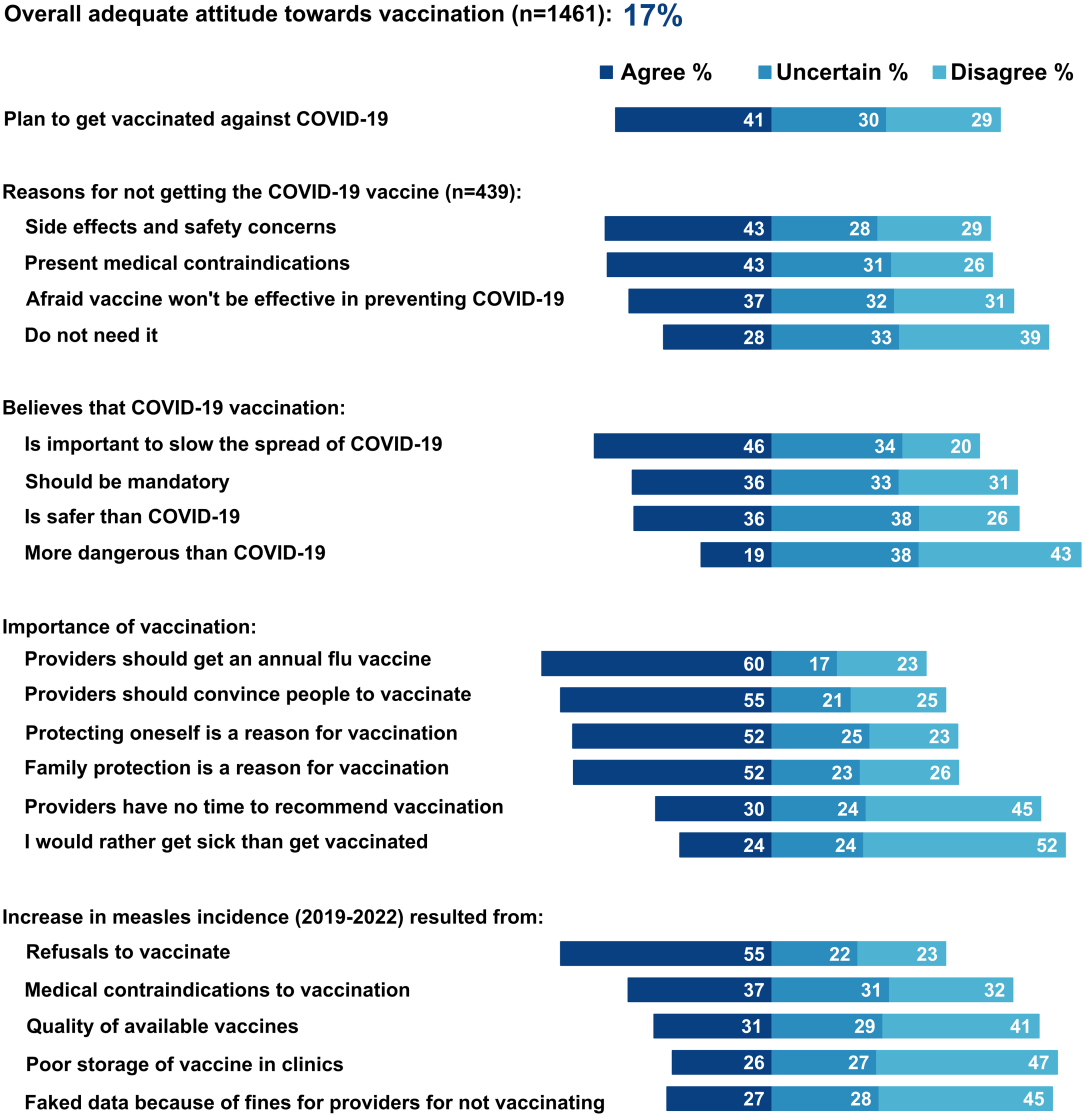
Attitudes toward vaccination among primary care providers, Aktobe, Shymkent, and Turkestan, Kazakhstan, 2021 (*N* = 1,461).

Among providers, 246 (17%) had adequate attitudes toward vaccination (scored above 70%), and 41% would get vaccinated against COVID-19 when it became available to them (Figure 1). Among those who refused vaccination (29%), the main reasons for refusing COVID-19 vaccination were side effects and safety concerns (43%), contraindications (43%), and belief that vaccines are not effective in preventing COVID-19 (37%). Half (46%) of providers believed that COVID-19 vaccination was important to slow transmission, and 19% believed that COVID-19 vaccination was more dangerous than getting COVID-19.

For other vaccines, 60% thought it was important for healthcare providers to be vaccinated against influenza, 55% would convince their patients to get vaccinated against childhood diseases or COVID-19, and 24% would rather get sick than get vaccinated themselves. Over half (55%) of providers believed that the 2019–2020 measles outbreak could largely be attributed to patient refusal to vaccinate (55%) and medical contraindications to vaccination (37%). Additionally, 27% believed that fines imposed on healthcare providers for not vaccinating their catchment population led to distorted vaccine coverage rates nationally.

**Figure 2:**
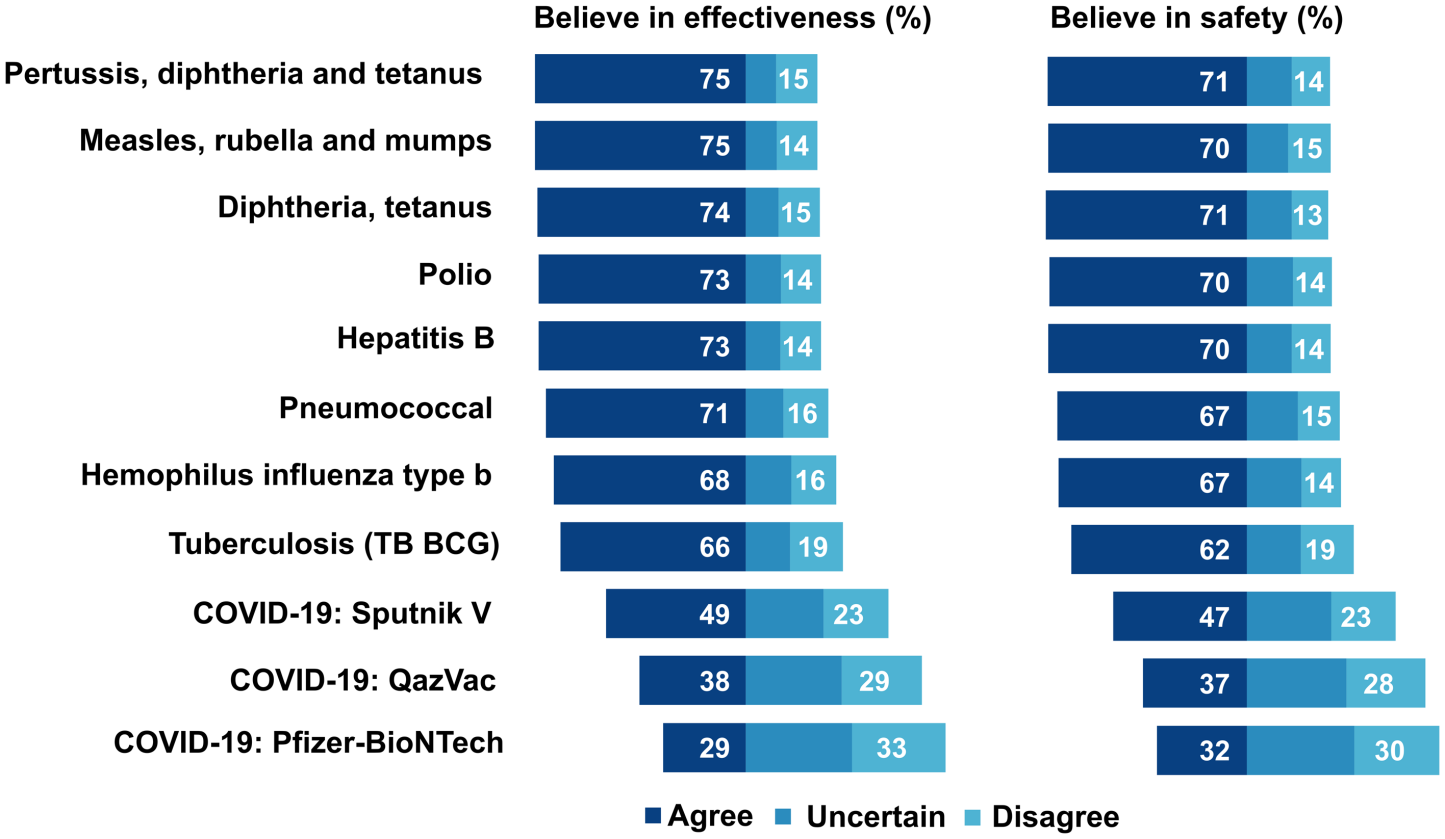
Attitudes toward safety and effectiveness of vaccines among primary care providers, Aktobe, Shymkent, and Turkestan, Kazakhstan, 2021 (*N* = 1,461).

Over 70% of providers believed in the safety and effectiveness of the majority of common childhood vaccinations (Figure 2). However, belief in the effectiveness and safety of COVID-19 vaccines was low. Belief in the effectiveness of Sputnik V was higher than that of the Pfizer-BioNTech COVID-19 vaccine (49 and 29%, respectively). Similarly, belief in safety was 47% for Sputnik V and 32% for the “Pfizer-BioNTech” COVID-19 vaccine.

### KAP and sociodemographic variables

**Figure 3:**
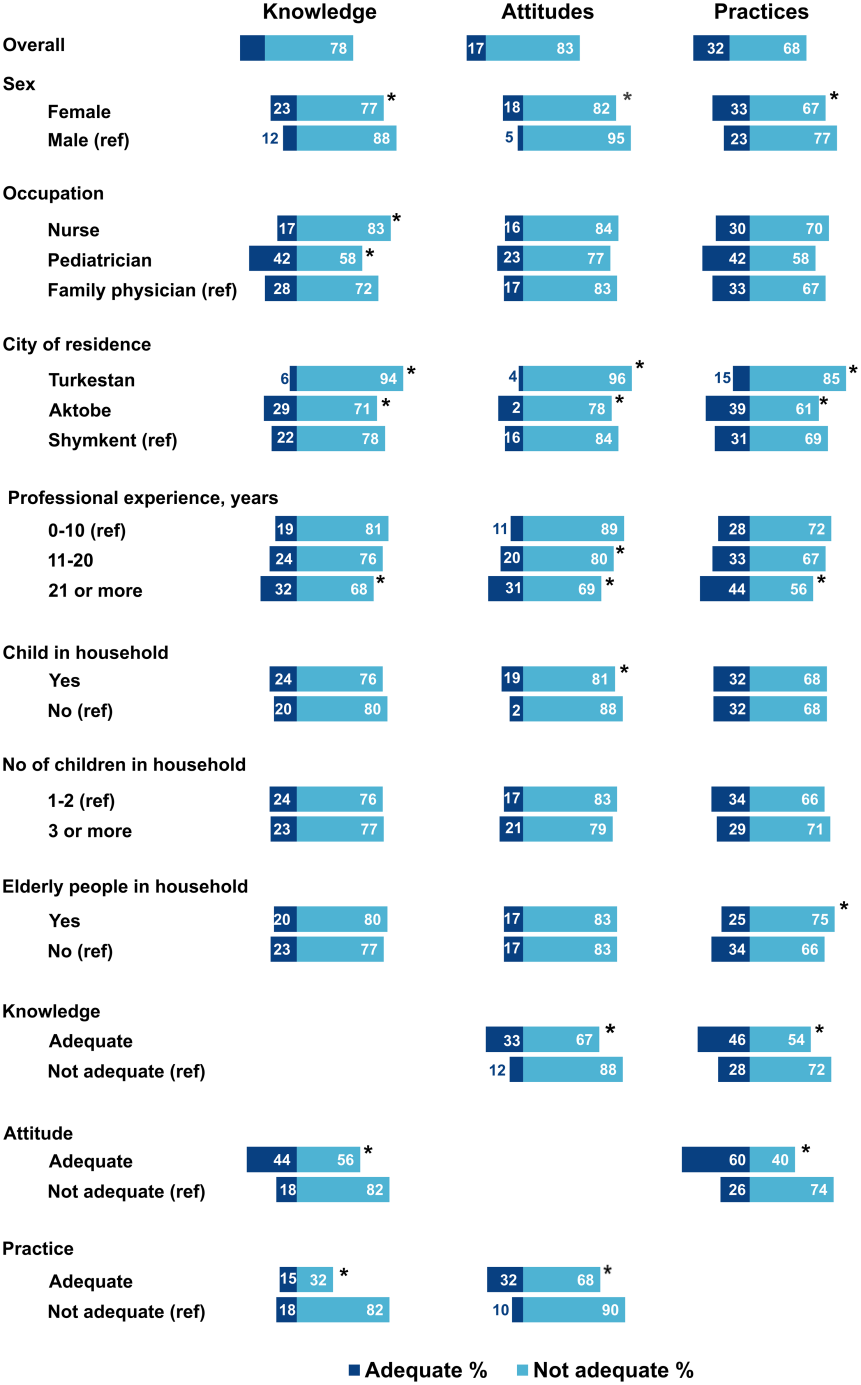
Adequate knowledge, attitudes, and practices toward vaccination among primary care providers, Kazakhstan, 2021 (*N* = 1,461). ^*^Significant difference, Chi-square value of *p* < 0.05; ref.: a reference group.

Adequate KAP was significantly higher (*p* < 0.05) among females compared to males, among those who worked in Aktobe and Shymkent compared to Turkestan, and among those who had over 20 years of professional experience compared to those who had less (Figure 3). A higher proportion of providers with children had adequate attitudes compared to those without children (19 vs. 12%, respectively; *p* = 0.002). Adequate practice was higher among those who did not have older adult in the household (34%) compared to 25% among whose household had older adult (*p* = 0.001).

### Relationship between knowledge, attitudes, and practices

**Figure 4:**
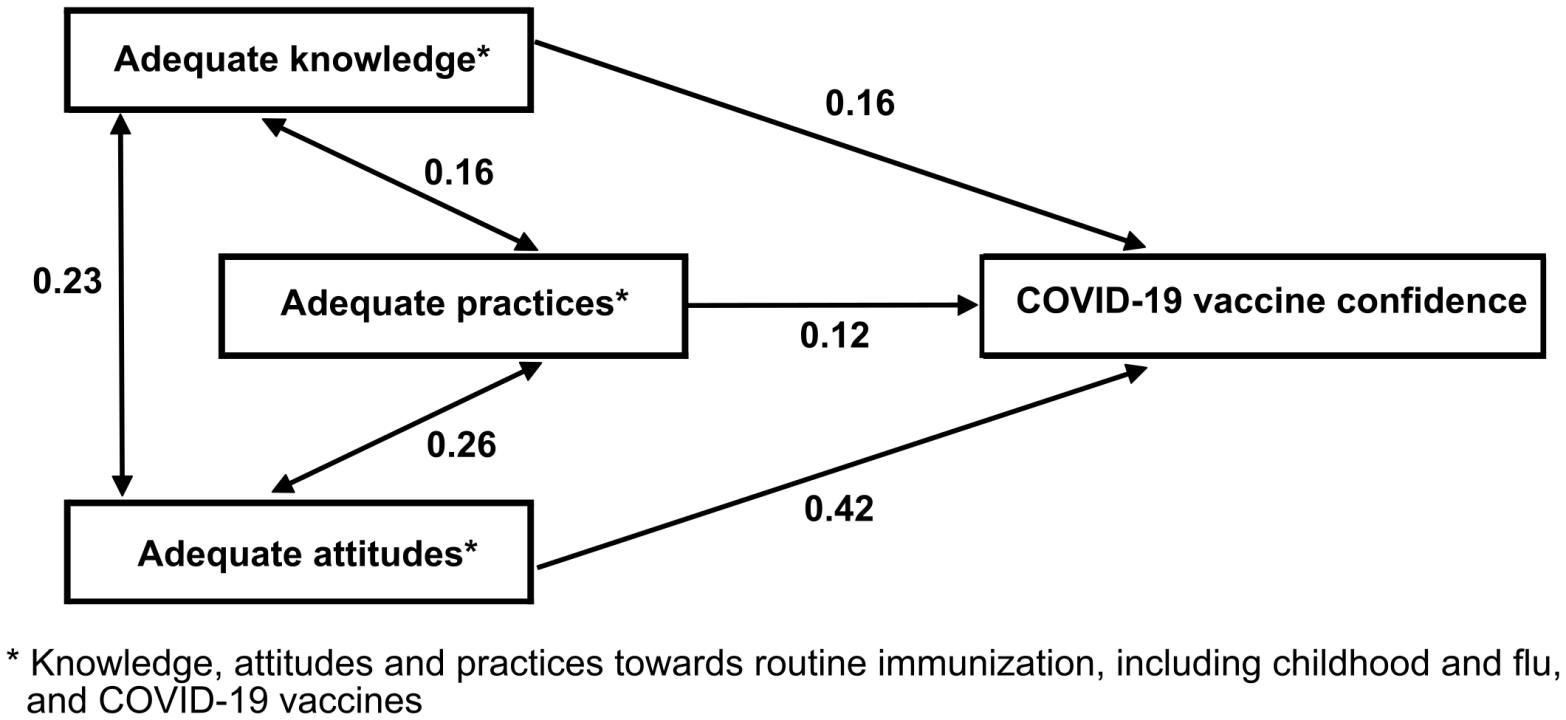
Correlation between adequate knowledge, attitude, and practice toward routine vaccination, including childhood, flu, and COVID-19 vaccine confidence, Kazakhstan 2021. Arrows indicate significant associations according to the multivariable regression analysis. Numbers over the arrows are Spearman correlation coefficients.

Adequate knowledge about vaccination in general was higher among providers with adequate attitudes (44%; *p* < 0.001; Figure 3). Similarly, adequate attitudes were significantly higher among those with adequate knowledge (33%; *p* < 0.001) and adequate practice (32%; *p* < 0.001). Just as adequate practice was higher among providers with adequate knowledge (46%) and adequate attitude (60%; *p* < 0.001). There was a moderate correlation (Spearman correlation coefficient of 0.42) between adequate attitudes and COVID-19 vaccine confidence (Figure 4). Additionally, the relationships between each domain of KAP are bidirectional, and each individual factor is positively associated with the other three (Table 4).

**Table 4:** The associations between adequate knowledge, attitude, and practice toward routine vaccination and COVID-19 vaccine confidence, Kazakhstan, 2021.

Outcome	Predictor	Correlation		Adjusted prevalence ratio^c^
		coefficient^a^	95%Cl^b^	
Vaccine confidence	Overall knowledge	0.16	(0.11–0.21)	**1.2 (1.0–1.4)**
Vaccine confidence	Overall attitude	0.42	(0.37–0.46)	**3.1 (2.7–3.6)**
Vaccine confidence	Overall practice	0.12	(0.07–0.17)	1.0 (0.9–1.1)
Overall knowledge	Overall attitude	0.23	(0.18–0.27)	**1.8 (1.5–2.2)**
Overall attitude	Overall knowledge	0.23	(0.18–0.27)	**1.9 (1.6–2.4)**
Overall knowledge	Overall practice	0.16	(0.11–0.21)	**1.3 (1.1–1.6)**
Overall practice	Overall knowledge	0.16	(0.11–0.21)	**1.3 (1.1–1.5)**
Overall attitude	Overall practice	0.26	(0.21–0.30)	**2.4 (1.9–2.9)**
Overall practice	Overall attitude	0.26	(0.21–0.30)	**1.9 (1.6–2.2)**

a Spearman correlation: no correlation 0.00–0.10, weak positive correlation 0.10–0.39, and 0.40–0.69 moderate positive correlation and > 0.70 strong positive correlation.

b CI, confidence interval.

c Values of *p* < 0.05 highlighted in bold.

### Bivariable analysis of factors associated with COVID-19 vaccine confidence

COVID-19 vaccine confidence was positively associated with adequate overall vaccine knowledge, knowledge of contraindications, adequate attitudes related to routine vaccination, belief in the effectiveness and safety of vaccines, and years of experience (Table 5). Providers who did not believe that COVID-19 vaccines were safer than getting COVID-19, those who did not think it was important to slow the spread of SARS-CoV-2 and those who did not think vaccines should be compulsory had lower COVID-19 vaccine confidence.

**Table 5:** Factors associated with COVID-19 vaccine confidence among primary health care providers in Kazakhstan, 2021: two multivariable Poisson regression models.

Characteristics		Model 1	Model 2 c
	cPR^a^ [95% CI]	aPR^b^ [95% CI]	aPR^b^ [95% CI]
Adequate knowledge (ref: not adequate)^c^	**1.7 [1.4–1.9]**	1.2 [1.0–1.4]^*^	
Knowledge about childhood and flu vaccination	**1.4 [1.2–1.7]**		1.1 [0.9–1.3]
Knowledge on contraindications for vaccination	**1.2 [1.0–1.4]**		0.9 [0.7–1.0]^*^
Knowledge about COVID-19 vaccination	**2.3 [2.0–2.7]**		1.2 [1.0–1.3]
Adequate attitude (*ref: not adequate*)	**3.4 [3.0–3.9]**	3.1 [2.7–3.6]^*^	
Effectiveness and safety of vaccines	**5.7 [4.2–7.9]**		2.8 [2.0–3.9]^*^
Immunization against measles	**1.2 [1.1–1.6]**		1.1 [0.9–1.2]
Disagree with the statement *(ref: agree/uncertain)*:			
Vaccination is important to slow the spread of COVID-19	**0.3 [0.2–0.4]**		0.6 [0.4–0.8]^*^
Vaccination should be mandatory	**0.2 [0.2–0.3]**		0.4 [0.3–0.5]^*^
I prefer to get sick rather than vaccinated?			1.2 [1.0–1.4]
Agree with the statement *(ref: disagree/uncertain)*:			
Providers should vaccinate against flu	**3.2 [2.6–4.1]**		1.5 [1.2–1.8]^*^
The role of a physician is to encourage timely vaccination			1.9 [1.6–2.4]^*^
I do not have time to convince people to vaccinate			0.8 [0.7–0.9]^*^
I trust QazVac vaccine			1.4 [1.1–1.8]^*^
I trust Sputnik V vaccine			1.3 [1.0–1.6]^*^
I trust BNT162b2 (Pfizer-BioNTech) vaccine			0.9 [0.8–1.1]
Adequate practice *(ref: not adequate)*	**1.4 [1.2–1.7]**	1.0 [0.9–1.1]	
Continuing education in vaccination *(ref: never)*	**1.3 [1.0–1.6]**		1.1 [0.9–1.4]
Professional experience *(ref: ≤10 years)*			
11–20	**1.4 [1.2–1.7]**	1.1 [0.9–1.4]	1.0 [0.8–1.2]
21 or more	**1.4 [1.1–1.7]**	0.9 [0.7–1.2]	0.8 [0.6–1.0]

^a^cPR, crude prevalence ratio. ^b^aPR, adjusted prevalence ratio [CI: confidence interval] (adjusted for age, city of residence, and child in a family). ^c^ref: reference group. ^*^Multivariable Poisson regression, significant difference, value of *p* < 0.05.

### Multivariable analysis of factors associated with COVID-19 vaccine confidence

In the reduced model (Model 1), COVID-19 vaccine confidence was associated with adequate attitude [adjusted prevalence ratio (aPR) = 3.1; 95% CI: 2.7–3.6] and adequate knowledge about routine immunization (aPR = 1.2; 95% CI: 1.0–1.4; Table 5; Supplementary Figure 2). In the fuller model (Model 2), factors associated with COVID-19 vaccination confidence were knowledge of COVID-19 vaccination (aPR = 1.2; 95% CI: 1.0–1.3), positive attitude toward the effectiveness and safety of vaccines (aPR = 2.8; 95% CI: 2.0–3.9), belief that it is important for primary care providers to vaccinate against flu (aPR = 1.5; 95% CI: 1.2–1.8), belief that the role of provider is to encourage timely vaccination (aPR = 1.9; 95% CI: 1.6–2.4), trust in the QazVac vaccine (aPR = 1.4; 95% CI: 1.1–1.8), and trust in the Sputnik V vaccine (aPR = 1.3; 95% CI: 1.0–1.6). Knowledge of contraindications (aPR = 0.9; 95% CI: 0.7–1.0), disagreement that vaccination is important to slow the spread of COVID-19 (aPR = 0.6; 95% CI: 0.4–0.8), that COVID-19 vaccination should be mandatory (aPR = 0.4; 95% CI: 0.3–0.5), and belief that providers have no time to convince people to vaccinate (aPR = 0.8; 95% CI: 0.7–0.9) were negatively associated with vaccine confidence.

## Discussion

### Key findings

Our study found that primary care providers in the cities of Shymkent, Turkestan, and Aktobe in Kazakhstan had low COVID-19 vaccine confidence during the time COVID-19 vaccines were first introduced in the country. Specifically, only 30% of providers had COVID-19 vaccine confidence. This proportion is within the range but low compared to the global average of 77% (range: 28–96%) across 35 studies of COVID-19 vaccine confidence among healthcare providers (37, 38). Additionally, one in three providers would refuse the vaccine, a proportion on the lower end of the range of those that would refuse a vaccine reported in the literature. By comparison, the pooled refusal rate from 51 studies among nurses worldwide was 21% (confidence interval: 17–27%) (39).

### Knowledge about vaccines and confidence

Because providers are at elevated risk for COVID-19, the high proportion of providers that would refuse the COVID-19 vaccine was concerning but not surprising (40). Low perceived trust in vaccine safety was the most important barrier to vaccination in our study, as it is in the literature (41, 42). We found that less than half of providers believed in safety and effectiveness of the most common COVID-19 vaccines available in Kazakhstan at that time. Low trust in COVID-19 vaccine safety and effectiveness could have been influenced by the lack of published data on the predominant COVID-19 vaccines available in Kazakhstan, Sputnik V, and QazVac (43–45). Most literature on COVID-19 vaccines was predominantly in English, as are most scientific publications, and therefore likely not easily accessible to the majority of healthcare providers in Kazakhstan (46), where 97% of the population speaks Russian and 80% speaks Kazakh (47).

Language is a critical barrier to accessing timely evidence-based medical and scientific literature (41). Proficiency in English is often required to access up-to-date vaccine research, guidelines, and training materials, which can influence medical practitioners’ understanding of and confidence in vaccines. Medical education and science in Kazakhstan have relied heavily on Russian-language Soviet textbooks without much if any, training on searching and using indexed peer-reviewed literature (48). Consistent with this is our finding that only approximately one in three providers access scientific literature on vaccines on a regular basis.

Similarly, language likely played an important role in our finding that almost twice as many participants trusted the Sputnik V vaccine safety and efficacy than trusted the Pfizer-BioNTech COVID-19 vaccines, even though the body of published evidence on safety and effectiveness was much larger for the latter. On the one hand, publications about Pfizer-BioNTech and other COVID-19 vaccines were almost exclusively in English. On the other hand, Russian-language misinformation was widely available in news and social media (49).

Our data also showed that one in two providers believed common myths about routine vaccinations; this gives further evidence that widespread Internet and social media vaccine misinformation is influencing healthcare provider knowledge about routine vaccines in Kazakhstan. For example, one in two providers still believed, incorrectly, that there is an association between vaccines and diseases such as autism and multiple sclerosis, even though hundreds of studies have been published (mostly in English) demonstrating otherwise. This is particularly worrisome because primary care providers in Kazakhstan are a trusted source on vaccination. Providers’ misconceptions may influence knowledge and attitude toward vaccination of the general population and there is rising vaccine hesitancy among the general population as well as high levels of common misperceptions such as belief that measles vaccination leads to autism or multiple sclerosis (50).

### Attitudes toward vaccines and confidence

Although providers with adequate knowledge about routine vaccines were 20% more likely to have COVID-19 vaccine confidence, knowledge had a weak positive correlation with attitudes, practice, and confidence. This finding suggests that knowledge alone is not sufficient to influence vaccine attitudes, confidence, and practice. As commonly reported in studies, attitudes related to vaccines play a larger role in influencing confidence and practice (51).

We found that adequate attitudes toward routine vaccines had a strong positive correlation with COVID-19 vaccine confidence. Additionally, providers with adequate attitudes toward routine vaccines were 210% more likely to have COVID-19 vaccine confidence. This finding is consistent with studies showing that healthcare providers with adequate attitudes toward childhood vaccination and acceptance of flu vaccination are more likely to have good attitudes related to COVID-19 vaccines and to get vaccinated (39, 52). This relationship is bidirectional, and people who are hesitant to childhood or flu immunization also tend to have poor attitudes toward COVID-19 vaccines (52–54).

Interestingly, in our multivariable model when controlling for measures of attitudes, we found that vaccine confidence was significantly lower for those with adequate knowledge of contraindications. This finding is in contrast to results from previous studies that demonstrate that high self-reported knowledge of contraindications is associated with higher rates of influenza vaccines (55). This finding demonstrates the importance of attitudes on confidence. We believe this finding could be explained by high knowledge of contraindications among hesitant providers who rely on their knowledge of contraindications to justify avoidance of vaccines that are mandated for healthcare providers, such as influenza, and subsequently COVID-19.

### Vaccine practice and confidence

Adequate practices related to vaccines had a weak positive association with adequate attitudes but no correlation with COVID-19 vaccine confidence. Although providers with adequate vaccine practices toward routine vaccines were more likely to have COVID-19 vaccine confidence in bivariable analysis, this relationship was not significant in multivariable models that adjusted for attitudes and knowledge.

Because prior studies have found that providers are a trusted source of vaccine information in Kazakhstan (11), the finding that two-thirds of providers felt it was difficult to speak to patients about vaccines was unexpected. Furthermore, over half of providers reported that they do not always feel confident answering patient questions about vaccines or patient concerns about vaccine side effects. These findings demonstrate a critical need for more comprehensive training programs focusing not only on the technical aspects of vaccination but also on strategies for communicating this information effectively (56). Health systems may need to allocate more resources to aid in vaccine-related discussions, including support staff, patient education materials, or even additional time for appointments.

While we did not ask about reasons for this difficulty in communicating about vaccines with patients, systemic barriers such as workload with 1,500–5,000 individuals in providers’ catchment areas, lack of time for detailed patient counseling, fines imposed for not vaccinating the population, or low reimbursement rates for vaccination services can hinder communication and vaccine advocacy.

Difficulty in communicating with patient populations about vaccines could also be related to providers not being up to date on recommendations. Our study found that a low proportion of primary care providers receive continuing education related to vaccination or independently study vaccine-related scientific literature. Policies and programs can help encourage proactive engagement with new knowledge (57). Systemic changes can help create a supportive environment for continuous learning, such as dedicated time for studying, promoting the value of continuous learning, and building a culture that encourages curiosity and staying up to date (58).

However, provision of continuous learning opportunities alone is not enough to change attitudes and behaviors. Learning opportunities can be reinforced using evidence-based approaches for behavior and attitude change such as nonjudgmental empathetic listening, personalized storytelling approach, and involvement of provider teams in communication with vaccine hesitant population (59). Also, providers can help change patient behavior and attitudes using evidence-based tools such as the American Medical Association STEPS Forward toolkit (60).

### Vaccine confidence and professional category

Similar to other studies, we did not find a significant association of vaccine confidence with age, years of professional experience, residence, presence of child or older adult people in the family, or continuing education in vaccination (61). However, we did find that KAP varied by medical profession, with pediatricians having a higher proportion of adequate KAP, followed by family physicians and nurses. Other studies have similarly found that nurses have lower knowledge and acceptance of COVID-19 vaccines than physicians (62, 63). These differences may be due to the level of training received in these specialties. In Kazakhstan, pediatricians may have higher KAP scores because they have more experience and training in routine immunization compared to other health professions. Family physicians in Kazakhstan have only recently become administrators of routine childhood vaccines (64).

### Study limitations

Our study is subject to at least three important limitations. First, our response rate was 42%. We have no information about nonresponders and cannot assess the level and direction of bias that this may have introduced in our results. Vaccine confidence in our study of 30% would range from 12 to 71% if all providers who refused would have participated and were either confident or not. Second, our study only included providers in three cities and may not represent the attitudes of healthcare providers across the entire country. Therefore, these results should not be extrapolated to all providers in Kazakhstan. Third, because participants were interviewed at their place of work, the results may have been subject to social desirability bias. The direction of this bias would likely have inflated our estimate of vaccine confidence. We attempted to mitigate this bias by making surveys anonymous and self-administered rather than using interviewer-assisted surveys. Fourth, we conducted this study during the initial stages of COVID-19 vaccine rollout, and COVID-19 vaccine confidence has grown since that time. However, our findings related to knowledge, attitudes, and practices toward routine childhood and influenza vaccines likely have not. These are likely entrenched KAP that would influence confidence of future vaccines.

### Study implications

We conducted this study during the early stages of COVID-19 vaccine rollout in Kazakhstan. COVID-19 vaccines have since become compulsory for healthcare providers and have resulted in high uptake of COVID-19 vaccines in providers. Whether the mandates resulted in increased COVID-19 vaccine knowledge and confidence is not known. However, it is unlikely that these mandates have had any impact on our findings of low levels of knowledge, attitude and practice toward routine vaccines. Changing the knowledge, attitude, practice and COVID-19 vaccine confidence of vaccine hesitant and refusing primary care providers often requires a multifaceted approach, including evidence-based education, dialog that addresses personal beliefs and attitudes, peer and community engagement, and systemic changes that make providing vaccines easier and more rewarding for healthcare providers. Given the vital role of healthcare providers in promoting vaccine uptake among the population, increasing KAP is vitally important to raise vaccine confidence in the general population.

## Conclusion

Our study uncovers critical areas for interventions to improve knowledge, attitudes, and practices related to routine immunizations and COVID-19 vaccine confidence among primary care providers in Kazakhstan. Low COVID-19 vaccine confidence was associated with inadequate overall knowledge about routine vaccines, negative overall attitudes, and misconceptions about the safety and effectiveness of both routine vaccines and COVID-19 vaccines. The strong correlation between attitudes toward routine vaccines and COVID-19 vaccine confidence underscores the importance of addressing vaccine hesitancy more broadly and not solely focusing on COVID-19. Our findings also highlight the need to ensure that relevant and reliable vaccine information is accessible in local languages. Policymakers can consider the findings of this study when designing and implementing vaccine strategies to healthcare providers in the vital role they have in fostering public trust in vaccination and achieving high coverage of COVID-19 vaccines and routine immunizations.

### Main message

Our study provides evidence of the need to improve knowledge, attitudes and practices related to routine immunizations and COVID-19 vaccination among primary care providers in Kazakhstan. Primary healthcare providers are ambassadors for vaccination and a trusted source of information on vaccination. Providers who are correctly informed and have positive views about vaccines, including COVID-19 vaccines, are able to navigate patient concerns. Kazakhstan has struggled with vaccine hesitancy with decreasing coverage of childhood immunizations and low uptake of COVID-19 vaccines. To our knowledge, this is the first study among healthcare providers in Central Asia that assesses levels of vaccine confidence and links it to knowledge, attitudes, and practices of routine immunizations. We interviewed 1,461 providers in 54 facilities. Participants included nurses, family physicians and pediatricians (65, 25, and 10%, respectively) whose duties include immunizations in 3 cities. We found that just one in three providers had confidence in COVID-19 vaccines. Additionally, less than one in three providers had adequate knowledge, attitudes or practices (22, 17, and 32%, respectively). Adequate knowledge and attitudes were positively correlated with COVID-19 vaccine confidence. Findings highlight the complex relationship between KAP of routine vaccines and COVID-19 vaccine confidence and the need to address hesitancy more broadly.

## Data availability statement

The raw data supporting the conclusions of this article will be made available by the authors, without undue reservation.

## Author contributions

DN, RH, LK, MS, AY, and AH: conceptualization. DN, RH, LK, MS, AH, SA, JN, and AY: methodology and writing—review and editing. DN, RH, SA, and LK: software, validation, and formal analysis. DN, LK, MS, and AH: investigation. DN, RH, MS, LK, AY, and JN: resources. DN, RH, AH, MS, and LK: writing—original draft preparation. DN, SA, and RH: visualization. AY, JN, and MS: supervision. MS and LK: project administration. All authors contributed to the article and approved the submitted version.

## Funding

Support for this project was provided by the United States Centers for Disease Control and Prevention, Central Asia Field Epidemiology Training Program (CDC Cooperative Agreement GH20-2108) in Almaty, Kazakhstan.

## Conflict of interest

The authors declare that the research was conducted in the absence of any commercial or financial relationships that could be construed as a potential conflict of interest.

## Publisher’s note

All claims expressed in this article are solely those of the authors and do not necessarily represent those of their affiliated organizations, or those of the publisher, the editors and the reviewers. Any product that may be evaluated in this article, or claim that may be made by its manufacturer, is not guaranteed or endorsed by the publisher.

## Author disclaimer

The findings and conclusions in this report are those of the author(s) and do not necessarily represent the official position of the United States Centers for Disease Control and Prevention.

## Footnotes

^1^^§^See, e.g., 45 C.F.R. part 46, 21 C.F.R. part 56; 42 U.S.C. ^§^241(d); 5 U.S.C. ^§^552a; 44 U.S.C. ^§^3,501 et seq.
