# Corrigendum: Factors associated with COVID-19 vaccine confidence among primary care providers in Kazakhstan, March–April 2021

**DOI:** 10.3389/fpubh.2023.1308374

**Published:** 2023-11-07

**Authors:** Dilyara Nabirova, Roberta Horth, Lena Kassabekova, Alden Henderson, Aizhan Yesmagambetova, Sevak Alaverdyan, J. Pekka Nuorti, Manar Smagul

**Affiliations:** ^1^Division of Global Health Protection in Central Asia, United States Centers for Disease Control and Prevention, Almaty, Kazakhstan; ^2^Central Asia Field Epidemiology Training Program, Asfendiyarov Kazakh National Medical University, Almaty, Kazakhstan; ^3^Health Sciences Unit, Faculty of Social Sciences, Tampere University, Tampere, Finland; ^4^Scientific and Practical Center of Sanitary-Epidemiological Examination and Monitoring, Branch of the National Center for Public Health, Almaty, Kazakhstan; ^5^Division of Global Health Protection, United States Centers for Disease Control and Prevention, Atlanta, GA, United States; ^6^Ministry of Healthcare, Astana, Kazakhstan; ^7^Manoogian Simone College of Business and Economics, American University of Armenia, Yerevan, Armenia; ^8^Infectious Diseases and Vaccinations Unit, Department of Health Security, Finnish Institute for Health and Welfare (THL), Helsinki, Finland

**Keywords:** COVID-19, vaccine confidence, primary care providers, attitude toward vaccination, childhood vaccines, Kazakhstan

In the published article, there was an error in Table 1 as published. Row and column percentages were inverted for the age group variable. The corrected Table 1 and its caption appear below.

**Table 1:** Perceptions toward COVID-19 vaccination among primary care providers, Aktobe, Shymkent, and Turkestan cities, Kazakhstan, 2021 (*N* = 1,461).

**Characteristics**	**Vaccine confidence *n* = 435 (30%^a^)**	**Vaccine hesitancy *n* = 587 (40%^a^)**	**Vaccine refusal *n* = 439 (30%^a^)**	**Total *N* = 1,461 (100%^b^)**	***P* value**
Occupation					
Nurse	276 (29)	382 (40)	293 (31)	951 (65)	0.202
Family physician	115 (32)	134 (37)	111 (31)	360 (25)	
Pediatrician	44 (29)	71 (47)	35 (23)	150 (10)	
Gender					
Male	24 (22)	44 (40)	42 (38)	110 (8)	0.076
Female	411 (30)	543 (40)	397 (29)	1351 (92)	
Age, years					
18–26	84 (25)	150 (44)	108 (32)	342 (23)	0.005
27–35	140 (29)	186 (38)	164 (33)	490 (34)	
36–66	204 (35)	231 (39)	154 (26)	589 (40)	
Missing	7 (18)	20 (50)	13 (33)	40 (3)	
City of residence					
Shymkent	151 (31)	190 (39)	148 (30)	489 (34)	< 0.001
Aktobe	232 (34)	266 (38)	194 (28)	692 (47)	
Turkestan	52 (19)	131 (47)	97 (35)	280 (19)	
Professional experience, years					
Median [Min, Max]	10 [1, 44]	7 [1, 46]	6 [1, 41]	8 [1, 46]	< 0.001
0–10	236 (26)	376 (41)	295 (33)	907 (62)	
11–20	97 (36)	88 (33)	82 (31)	267 (18)	
21 or more	102 (36)	123 (43)	62 (22)	287 (20)	
Child in the family					
Yes	324 (32)	397 (39)	300 (29)	1021 (70)	0.043
No	111 (25)	190 (43)	139 (32)	440 (30)	
Number of children in the family					
Median [Min, Max]	2 [1, 6]	2 [1, 7]	2 [1, 6]	2 [1, 7]	0.938
1–2	168 (31)	219 (40)	157 (29)	544 (37)	
3 or more	156 (33)	178 (37)	143 (30)	477 (33)	
Missing	111 (25)	190 (43)	139 (32)	440 (30)	
Elderly people in the household					
Yes	104 (32)	122 (38)	99 (30)	325 (22)	0.486
No	331 (29)	465 (41)	340 (30)	1136 (78)	

^a^Row percent; ^b^Column percent.

In the published article, there was an error.

A correction has been made to Results, *Sociodemographic data and vaccine confidence*, paragraph 2. This sentence previously stated:

“Confidence was 47% among providers 36 years old and above and 19% among those 18–26 years old.”

The corrected sentence appears below:

“Confidence was 35% among providers 36 years old and above and 25% among those 18–26 years old.”

The authors apologize for these errors and state that this does not change the scientific conclusions of the article in any way. The original article has been updated.

