# Supplementary material for: Factors associated with COVID-19 vaccine confidence among primary care providers in Kazakhstan, March–April 2021
Source: Front Public Health. 2023 Sep 7;11:1245750. doi: 10.3389/fpubh.2023.1245750 (PMC10517263; doi:10.3389/fpubh.2023.1245750)
Supplement: Supplementary file 1 [file Table_1.docx]

**Supplementary Table 1A |** Knowledge questions used for the study

| **Domain** | **Questions** |
| --- | --- |
| Contraindications | Temporary contraindications to immunization for all vaccines: |
|  | Fever, Yes (T)^a^ |
|  | Prematurity^b^, Yes (T) |
|  | Birth weight < 2500 gram, No (T) |
|  | Uncontrolled seizures or progressive encephalopathy, Yes (T) |
|  | Steroid use for live vaccines, Yes (T) |
|  | Antibiotics use, No (T) |
|  | Acute diseases, regardless of temperature, Yes (T) |
|  | 2-month-old babies that are breastfed, No (T) |
|  | Permanent contraindications to immunization for all vaccines: |
|  | Severe allergic reaction to the previous dose, Yes (T) |
|  | Malignant neoplasms with active chemotherapy, Yes (T) |
|  | Immunodeficiency diseases for live vaccines, Yes (T) |
|  | People, who were vaccinated against diphtheria or who had a toxic form of diphtheria, should be revaccinated 6 months after they have had a disease, Yes (T) |
| Measles | Possible side effects of measles vaccine: |
|  | Brain and spinal cord inflammation (encephalomyelitis), Yes (T) |
|  | Elevated temperature, Yes (T) |
|  | Urticaria, Yes (T) |
|  | Mild local reaction such as redness and soreness at the injection site, Yes (T) |
|  | Thrombocytopenia, Yes (T) |
| Childhood & flu vaccination | Simultaneous administration of several vaccines overloads the immune system, No (T) |
|  | Current scientific evidence supports a link between vaccines and diseases like autism and multiple sclerosis, No (T) |
|  | Children who had pertussis may be vaccinated later with a vaccine containing the pertussis component, Yes (T) |
|  | Pertussis vaccine causes Sudden Infant Death syndrome, No (T) |
|  | Should the Hepatitis B (HBV) immunization schedule be restarted if one of doses was missed or delayed? No (T) |
|  | The flu vaccine causes the flu, No (T) |
| COVID-19 vaccination | Sputnik V is a vector-based COVID-19 vaccine, Yes (T) |
|  | The efficacy of Sputnik V vaccine against COVID-19 exceeds 90%, Yes (T) |
|  | Sputnik V vaccine induce sufficient humoral and cellular immune responses, Yes (T) |
| a T: True; b for Bacille CalmetteGuérin (BCG) and hepatitis B vaccines | |

**Supplementary Table 1B** | Attitude questions used for the study

| **Domain** | **Questions** |
| --- | --- |
| **Effectiveness, safety of childhood vaccines and refusals to vaccinate** | Fully believe in effectiveness of the following vaccines: |
|  | Pertussis, diphtheria and tetanus, Agree/fully agree |
|  | Measles, rubella and mumps, Agree/fully agree |
|  | Diphtheria, tetanus, Agree/fully agree |
|  | Polio, Agree/fully agree |
|  | HBV, Agree/fully agree |
|  | Pneumococcal infection vaccine, Agree/fully agree |
|  | Haemophiles influenza type b, Agree/fully agree |
|  | TB vaccine, Agree/fully agree |
|  | Fully believe in safety of the following vaccines: |
|  | Pertussis, diphtheria and tetanus, Agree/fully agree |
|  | Measles, rubella and mumps, Agree/fully agree |
|  | Diphtheria, tetanus, Agree/fully agree |
|  | Polio, Agree/fully agree |
|  | HBV, Agree/fully agree |
|  | Pneumococcal infection vaccine, Agree/fully agree |
|  | Haemophiles influenza type b, Agree/fully agree |
|  | TB vaccine, Agree/fully agree |
|  | Believe that people refuse to vaccinate due to: |
|  | Fear of unfavorable response, Agree/fully agree |
|  | Failure to perceive the disease as a serious threat, Agree/fully agree |
|  | Lack of trust in effectiveness of vaccination, Agree/fully agree |
|  | True contraindications to vaccination due to medical reasons, Agree/fully agree |
| **Measles incidence increase in 2019-2020** | What do you believe to be the reason for an increase of measles incidence rates in RK in 2018 – 2020: |
|  | Vaccination refusal, Agree/fully agree |
|  | Medical contraindicators for vaccination in a large number of cases, Agree/fully agree |
|  | Vaccine quality, Disagree/fully disagree |
|  | Failure to comply with storage conditions in a clinic, Disagree/fully disagree |
|  | Failure to comply with transportation of vaccine to the clinic, Disagree/fully disagree |
|  | Insufficient number of healthcare workers in a health facility to actively telephone and invite people to get vaccinated, Disagree/fully disagree |
|  | A heavy workload of primary care providers at the catchment area apart from the time required to convince people to get vaccinated, Disagree/fully disagree |
|  | Reason for high measles vaccination rates in RK in 2018-2019: |
|  | It is a true high vaccination coverage of an appropriate group, Agree/fully agree |
|  | Healthcare workers are penalized for cases of vaccination refusal. Therefore, healthcare workers are forced to register cases of vaccination administration that never took place. Disagree/fully disagree |
|  | Healthcare workers are penalized for cases of vaccination refusal. Therefore, healthcare workers are forced to issue withdrawals from vaccination for medical reasons, which in reality do not exist. Disagree/fully disagree |

**Supplementary Table 1C** | Attitude questions used for the study

| **Domain** | Questions |
| --- | --- |
| **Importance of vaccination** | It is important to encourage all healthcare workers to get an annual influenza vaccine, Agree/fully agree |
|  | Agree with the statement: ‘I prefer to get sick rather than vaccinated’? Disagree/fully disagree |
|  | Do you think that the role of a physician is to encourage timely vaccination? Agree/fully agree |
|  | Agree with the statement: ‘I don’t have enough time to call people and convince them of the need to get vaccinated’?, Disagree/fully disagree |
|  | Believe that penalties imposed on providers for failure to vaccinate the population distort a true picture of vaccination in the country, Disagree/fully disagree |
|  | Believe that protection of a family is a reason for vaccination, Agree/fully agree |
|  | Believe that polio immunization should be provided even though polio is now a rare disease, Agree/fully agree |
|  | Believe that self-protection is the reason for vaccination, Agree/fully agree |
|  | Believe that protection of patients is the reason for vaccination, Agree/fully agree |
| **COVID-19 vaccination** | Fully believe in effectiveness of the following vaccines: |
|  | Sputnik V COVID-19 vaccine, Agree/fully agree |
|  | QazVac COVID-19 vaccine, Agree/fully agree |
|  | BNT162b2 (Pfizer-BioNTech) COVID-19 vaccine, Agree/fully agree |
|  | Fully believe in safety of the following vaccines: |
|  | Sputnik V COVID-19 vaccine, , Agree/fully agree |
|  | QazVac COVID-19 vaccine, Agree/fully agree |
|  | BNT162b2 (Pfizer-BioNTech) COVID-19 vaccine, Agree/fully agree |
|  | Trust to COVID-19 vaccines |
|  | I trust the QazVac vaccine, Agree/fully agree |
|  | I trust the Sputnik V vaccine, Agree/fully agree |
|  | I trust Modena vaccine, Agree/fully agree |
|  | I trust BNT162b2 (Pfizer-BioNTech) vaccine, Agree/fully agree |
|  | Believe that vaccination should be mandatory, Agree/fully agree |
|  | Vaccination against COVID-19 is >dangerous than COVID-19, disagree/fully disagree |
|  | Reasons for refusing to vaccinate against COVID-19: |
|  | Fear of adverse effects and safety, Disagree/fully disagree |
|  | Concerns that it will not be effective in preventing COVID-19, Disagree/fully disagree |
|  | I don’t need it, Disagree/fully disagree |
|  | It will be too expensive, Disagree/fully disagree |
|  | Medical contraindications, Agree/fully agree |
|  | Vaccination is important to slow down the spread of COVID-19 in the population, Agree/fully agree |

**Supplementary Table 1D** | Practice questions used for the study

| **Domain** | **Questions** |
| --- | --- |
|  |  |
|  | Rely on own judgment, not manufacturer's recommendations when administering vaccines, Never |
|  | Rely on colleagues' opinions when administering vaccinations and working with those who refuse, Never |
|  | Recommend immunization according to friends and family members, Always/sometimes |
|  | Find it difficult to start a conversation about vaccination with parent or patient, Never |
|  | Confident when answering patients’ questions about vaccines, Always/sometimes |
|  | Comfortable addressing patient's vaccine side effects concerns, Always/sometimes |
|  | Try to reduce parents’ stress by dividing a conversation about immunization into several stages/visits, Always/sometimes |
| **Continuing education** | Receive continuing education in vaccination, Always/sometimes: |
|  | Independently study scientific publications, Always/sometimes: |
|  | Attend workshops and trainings provided by healthcare organizations, Always/sometimes: |
|  | Attend online workshops and seminars, Always/sometimes: |
|  | Rise your vaccination knowledge and qualification level on a regular basis, Always/sometimes: |
|  | Quarterly, Always/sometimes: |
|  | Annually, Always/sometimes: |
|  | Less than once every 3 years, Always/sometimes: |
|  | Less than once every 5 years, Always/sometimes: |

**Supplementary Table 2 |** Multivariable Poisson regression models with outcome of COVID-19 vaccine confidence.

| **Characteristics** | **Model 1** | **Model 2** |
| --- | --- | --- |
| Sociodemographics (Confounders) | Age, city of residence, professional experience, child in the family | Age, city of residence, professional experience, child in the family |
| **K**nowledge toward | Overall knowledge | Childhood & flu vaccination,  Contraindications for vaccination,  COVID-19 vaccination |
| **A**ttitude toward | Overall attitude | Attitude –   - Effectiveness and safety of vaccines, - Importance of vaccination, - COVID-19 vaccination, - Immunization against measles,   Belief that   - COVID-19 vaccination - Is more dangerous than COVID-19, - Is important to slow the spread of COVID-19 and - Should be mandatory, - That it is important for PCW to vaccinate against flu |
| **Pr**actice toward | Overall practice | Continuing education in vaccination and  Overall practice toward vaccination |
