# Supplementary material for: Factors associated with COVID-19 vaccine confidence among primary care providers in Kazakhstan, March–April 2021
Source: Front Public Health. 2023 Sep 7;11:1245750. doi: 10.3389/fpubh.2023.1245750 (PMC10517263; doi:10.3389/fpubh.2023.1245750)
Supplement: Supplementary file 2 [file Image_1.pdf]

Supplementary Figure 1 | Factors associated with COVID-19 vaccine confidence among primary health care providers in Kazakhstan, 2021: two multivariable Poisson regression models

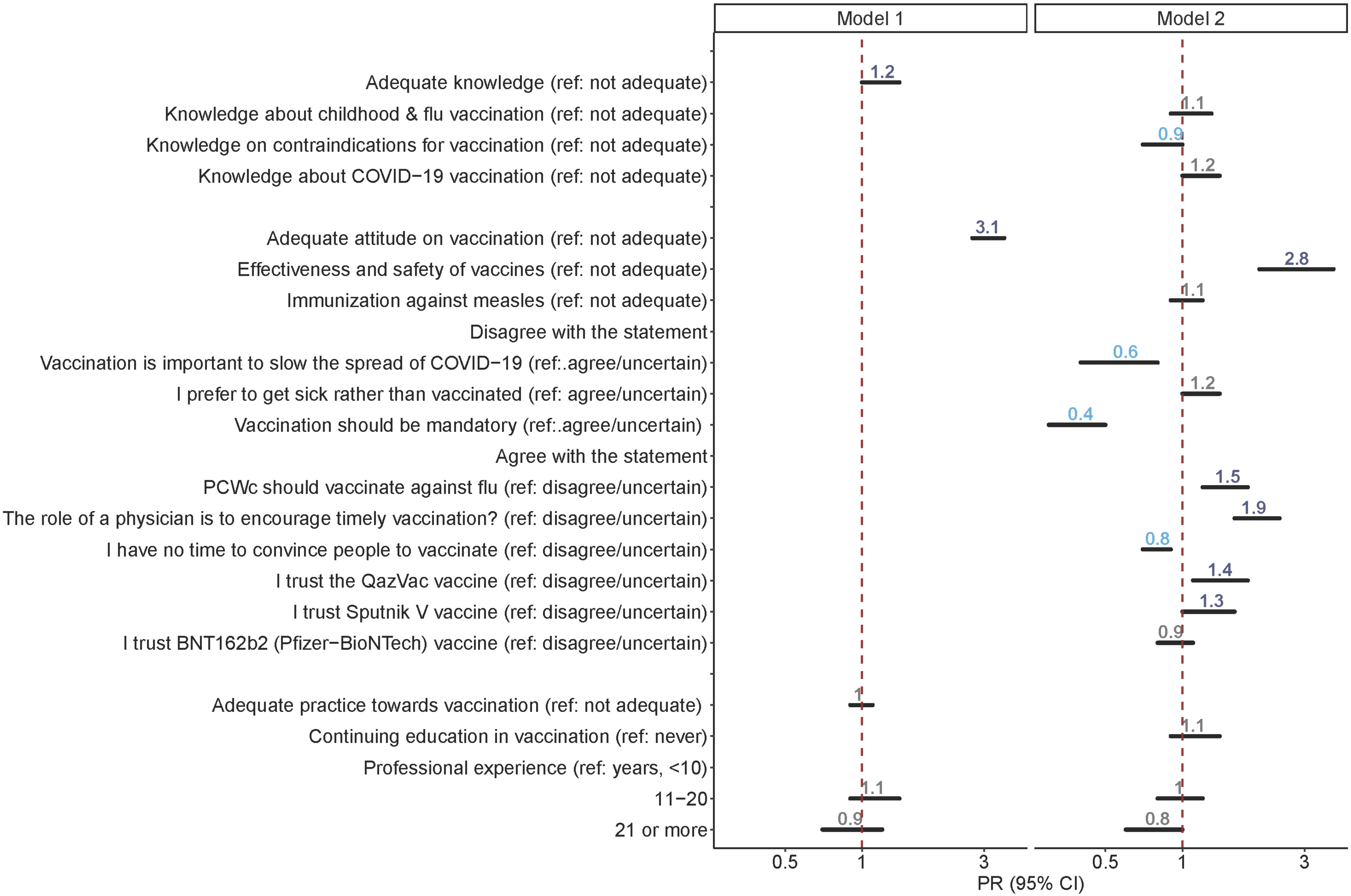

PR: adjusted prevalence ratio (adjusted for age, city of residence and child in a family) - Multivariable Poisson regression, **significant difference, p value <0.05;** ref: reference group; PCW: primary care worker
