# Supplementary material for: Factors associated with COVID-19 vaccine confidence among primary care providers in Kazakhstan, March–April 2021
Source: Front Public Health. 2023 Sep 7;11:1245750. doi: 10.3389/fpubh.2023.1245750 (PMC10517263; doi:10.3389/fpubh.2023.1245750)
Supplement: Supplementary file 3 [file Image_2.pdf]

Supplement Figure 2 | Power calculation for study sample size

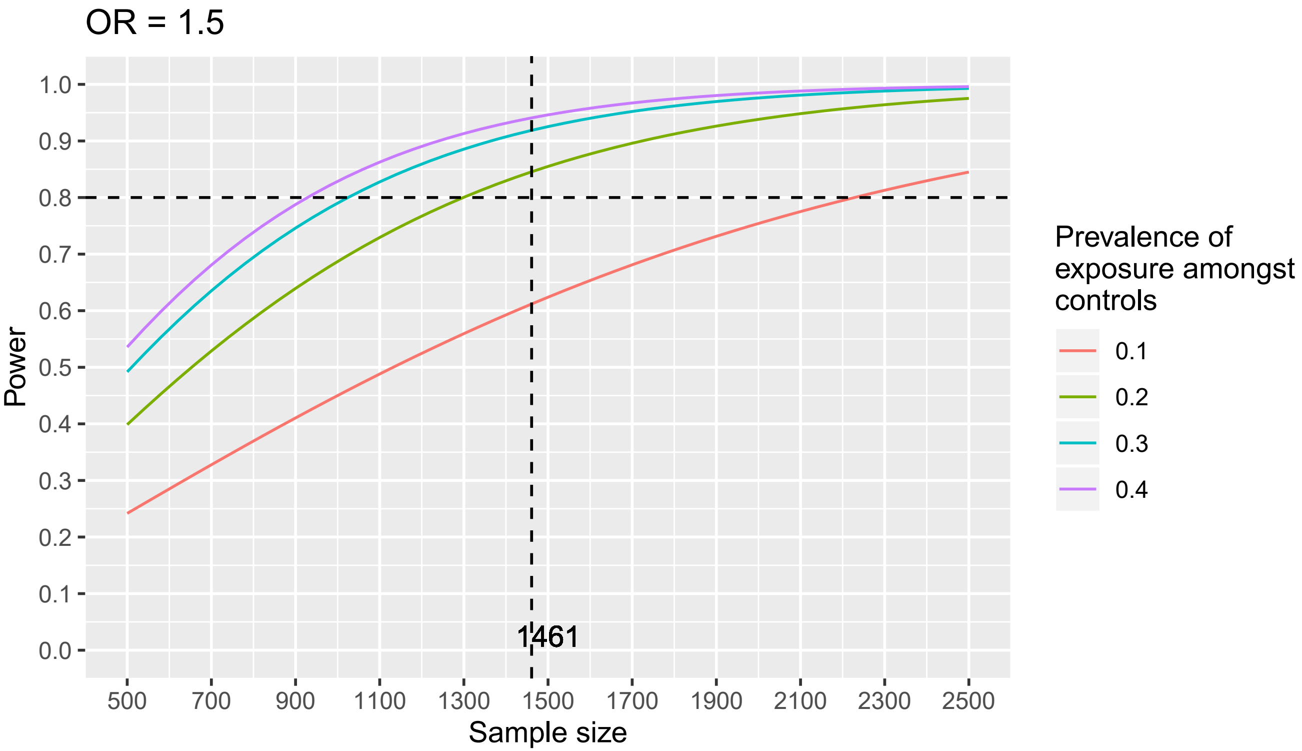

Note: The study sample size was 1461. In the power calculation, we are assuming an odds ratio of 1.5, 95% confidence level, and 435/1026 ratio of comparable group sizes (adequate to not adequate KAP).
